# ACTN3 Gene and Susceptibility to Sarcopenia and Osteoporotic Status in Older Korean Adults

**DOI:** 10.1155/2017/4239648

**Published:** 2017-05-24

**Authors:** Jinkyung Cho, Inhwan Lee, Hyunsik Kang

**Affiliations:** College of Sport Science, Sungkyunkwan University, Suwon, Republic of Korea

## Abstract

**Background:**

Little information is available about molecular markers for sarcopenia and osteoporosis in Asian populations.

**Objective:**

This study investigated the association of the* ACTN3* polymorphism with sarcopenia and osteoporotic status in older Korean adults.

**Methods:**

Older Korean 62 men and 270 women (mean age 73.7 ± 6.6 years) participated in this study. Body mass index, percent body fatness, appendicular skeletal muscle mass, and bone mineral density of the lumbar spine, femur, and total body were analyzed with dual-energy X-ray absorptiometry.* ACTN3* R/X genotyping was determined using TaqMan probes.

**Results:**

Determination of odds ratios (ORs) and 95% confidence intervals (CIs) using binary logistic regression analyses showed that XX homozygotes were at a significantly higher risk of sarcopenia (OR = 2.056, 95%  CI = 1.024–4.127, *p* = 0.043) and osteoporosis (OR = 2.794, 95%  CI = 1.208–5.461, *p* = 0.016) than RR homozygotes (reference group, OR = 1). The OR of XX homozygotes for having sarcopenia remained significant (OR = 2.237, 95%  CI = 1.044–4.836, *p* = 0.038) after adjustments for age, gender, body fatness, and serum vitamin D. The OR of XX homozygotes for having osteoporosis was no longer significant (OR = 2.682, 95%  CI = 0.960–7.942, *p* = 0.075) after adjustments for the covariates.

**Conclusion:**

Our findings suggest that the* ACTN3* R577X genotype may influence decline in muscle and bone health phenotypes in older Korean adults.

## 1. Introduction

Sedentary aging involves structural and functional changes that are conducive to mobility limitations, falls, fractures, and disability [1]. Many of these physical impairments are due to a progressive decline in lean body mass (LBM), a gradual deterioration in bone mineral density (BMD), or both. Therefore, decline of LBM in conjunction with low BMD is the most clinically useful phenotype for frailty. These features are closely associated with use of a cane or walker [2], a history of falling [3], and self-reported difficulty with gait and daily activities [4].

The global population aged 60 years or older has increased in nearly all countries in conjunction with decreased mortality and declining fertility. Due to the same reasons in conjunction with increasing average life expectancy at birth, Korea is moving toward an aged society at the fastest pace in the world. In 2010, Koreans aged 65 years and older reached 11% in the population, and the number is projected to increase to 24.3% in 2030 and reach 37.4% in 2050 [5]. This rapid population aging in Korea has raised public health concerns such as sarcopenia and osteoporosis. Sarcopenia and osteoporosis are two closely linked disorders, especially affecting elderly persons, and represent a major public health concern in Korea [6]. Thus, research regarding the impact of the two disorders is essential for the development of public health programs for the increasingly elderly Korean population.

Individual differences in LBM and BMD are primarily determined by differences in genetic makeup [7]. Heritability studies in humans found overall genetic contributions of up to 45–76% for fat free mass [8] and 65% for muscle strength [9]. Twin and family studies have demonstrated that BMD is a quantitative trait determined by genetic factors with an influence ranging from 50 to 85% [10, 11]. Genetic factors likely contribute to susceptibility to sarcopenia and osteoporotic status [9, 12]. Thus, their elucidation could be of paramount importance for designing more effective treatment strategies to manage structural and functional changes of the musculoskeletal system with aging.

The molecular bases of sarcopenia and osteoporotic status have been actively explored to identify candidate genes; however, these identifications have been at best tentative and occasionally inconsistent. A few genes have been established as contributing to variation in LBM [13, 14] and BMD [15]. These genes include angiotensin 1 converting enzyme 1, alpha-actinin 3* (ACTN3)*, myostatin, ciliary neurotrophic factor, and vitamin D receptor. Among the well-known candidate genes for physical fitness and/or performance is the* ACTN3 *gene. The human* ACTN*3 gene encodes *α*-actinin-3 protein, which is specifically expressed in fast glycolytic Type IIx fibers and 50% of fast oxidative Type IIa fibers [16], and is related to the generation of contractile force at high speeds [17]. A common genetic variation in this gene causes replacement of an arginine (R) with a premature stop codon (X) at amino acid 577 [18].

In previous animal studies involving* Actn3* knockout mouse, MacArthur et al. [17, 18] and Seto et al. [19, 20] showed that homozygote for 577X is detrimental for power and/or strength and beneficial for endurance performance. In addition, Lee et al. [21] in a recent review discussed three biological pathways by which alpha-actinin-3 deficiency alters muscle function in favor of endurance performance while sacrificing muscle strength and/or power, a shift in the fast fiber properties towards those of slow fibers (structural) and accompanying higher activity of oxidative enzymes (metabolic) and higher baseline calcineurin activity (signaling). Likewise, experimental evidences showing the involvement of alpha-actinin-3 deficiency in reduced BMD have also been reported from human [22] and animal studies [23].

Unlike for physical fitness and performance, however, published data on* ACTN3* genotypes and LBM and BMD phenotypes in older adults are controversial and are mainly from Caucasian populations [24–26]. Few studies have investigated the association of* ACTN3* genotypes with sarcopenia and osteoporotic status in Asian populations. This study investigated the association between* ACTN3* genotype and susceptibility to sarcopenia and osteoporotic status in older Korean adults.

## 2. Methods

### 2.1. Participants and Procedure

A total of 332 older adults were recruited via advertisements (local newspapers and flyers) in local community centers in the city of Suwon located in the Northwestern Gyeonggi Province, Republic of Korea, between December 2014 and August 2016. Eligibility criteria included men (*n* = 62) or women (*n* = 270) aged 65 years or older; no self-reported difficulty walking; no difficulty performing basic activities of daily living; no reported cane use, walker, crutches, or other special equipment to get around; no active treatment for cancer in the prior three years; and no enrollment in a lifestyle intervention trial. All participants had a homogeneous geographical ancestry wherein both parents were from the Korean Peninsula. The Sungkyunkwan University Institutional Review Board, in accordance with the World Medical Association Declaration of Helsinki, reviewed and approved the study protocol. Informed consent was obtained from all participants prior to the study.

Data were collected during two separate visits. During the first visit, demographic characteristics and general health status were assessed with a standardized self-administered questionnaire modified from the ACSM Guidelines for Exercise Testing and Prescription [27]. Interviews were conducted by geriatric nurses. During the second visit, height was assessed using a measuring tape with the participant standing with the back of the head, scapulae, buttocks, and heels in contact with a vertical board. Body weight was measured using a portable digital scale, after removing shoes and wearing only light clothing (Jenix, Seoul, Korea). BMI was calculated by dividing body weight by height squared (kg/m^2^). Fasting serum and whole-blood samples were collected for assessment of vitamin D and* ACTN3* genotyping, respectively. Bone mineral density and LBM were measured as described below.

### 2.2. Determination of Sarcopenia and Osteoporotic Status

DEXA measurements were made using the Lunar DPX Pro total body pencil beam densitometer (GE Lunar, Madison, WI, USA), while only exposing subjects to one scan. Before participation, the subjects were asked to disclose information about weight and pregnancy status, as both could exclude them from having the scan performed due to safety and health reasons. Subjects were also instructed to disclose any information about implanted metal that could affect the analysis of the scan. The subjects were contained within the imaging field of the DEXA scanner. Additionally, the subjects were generally healthy, and weight was stable at the time of measurement. All the subjects met the inclusion criteria. The exclusion criteria included current patient's report of any disease that affects bone metabolism, including hyperthyroidism, hyperparathyroidism, chronic renal failure, bronchial asthma, rheumatoid arthritis, and cancer, and objects which are being taken, such as glucocorticoids or thyroid hormone.

One day before the DEXA scan, the subjects were advised not to participate in any strenuous physical activity, to avoid any caffeine-containing beverages, and to drink water as usual. On the morning of the exam, the subjects arrived at the laboratory in 8–10-hr overnight fasting. The subjects were asked to remove their shoes, all objects from their pockets, and any jewelry or metal before being scanned. Then the subjects lied supine with the face and torso facing up in the center of the DEXA platform with their entire body within the field of the scan as outline on the DEXA platform. The arms rested by the subject's side with palms of the hands resting on the table top and fingers slightly separated, but the thumbs were not placed under the buttocks. X rays were then delivered at two energies from a low current X-ray located beneath the DEXA machine. The two X-ray energies were then passed through the subject, posterior to anterior, and collected in the detector arm above. The duration of the scan ranged from 10 to 15 minutes depending on the thickness of the subject. The effective dosage of the DEXA scan is 0.001 mSv (from the manufacturer's technical data), which is comparable to natural background radiation for 3 hours. DEXA measurement methods and validation have been reported elsewhere [28].

Appendicular skeletal muscle mass (ASM) was obtained by adding the LBM of the four limbs. The skeletal mass index (SMI) was defined as ASM/height^2^. We used the Asian Working Group for Sarcopenia (AWGS) to determine the prevalence of sarcopenia in this study sample [29]. The AWGS adopts the cutoff value by using the value two standard deviations below the mean for a young reference group, which is 7.0 and 5.4 kg/m^2^ for men and women, respectively.

BMDs at the lumbar spine (L1–L4), femur (neck and total), and total body were measured by the DEXA scan. Results of the bone densitometry were expressed in g/cm^2^ and *T*-score, as calculated by the device. Osteopenia (low BMD) was defined as a *T*-score at the femoral neck of between −1.0 standard deviation (SD) and −2.5 SD below the young adult mean (*T*-score < −1 and >−2.5). Osteoporosis was defined as a *T*-score at the femoral neck of 2.5 SD or more below the young adult mean (*T*-score ≤ −2.5) according to World Health Organization criteria [30]. *T*-score reflects a value compared with that of persons matched for age and sex in Korean general population aged 20–40 years old (from the manufacturer's technical data).

### 2.3. Serum Vitamin D and* ACTN3* Genotyping

Serum vitamin D levels were determined using LIAISON 25(OH) Vitamin D TOTAL Assays, which are direct competitive chemiluminescence immunoassays for human serum that use the DiaSorin LIAISON automated analyzer (Italy, DiaSorin SpA). Intra-assay coefficients of variation for serum vitamin D were 3 to 6% and interassay coefficients were 7 to 11%.

Blood samples for genotyping were collected in tubes containing disodium-EDTA as an anticoagulant and stored at −70°C until extraction. Total DNA was extracted from whole-blood samples using QIAmp DNA Mini Kits (QIAGEN, Hilden, Germany), following the kit instructions. The* ACTN3* R577X (rs1815739) polymorphism was genotyped using TaqMan real-time PCR (Applied Biosystems, Foster City, CA, USA), according to the protocol provided by the manufacturer. PCR reactions contained 10 ng genomic DNA, 12.5 *μ*l TaqMan Genotyping Master-mix (2x), 0.625 *μ*l Custom TaqMan genotyping assay mix (20x), and various amount of DNase-free, RNasefree water so that the final PCR volume was brought up to 25 *μ*l per reaction. The amplification protocol (95°C for 10 min followed by 40 cycles at 95°C for 15 s and 60°C for 1 min) was performed using Applied Biosystems 7500 instruments (Applied Biosystems).

### 2.4. Statistics

Haploview 4.1 (https://www.broadinstitute.org/haploview/haploview) was used to check Hardy-Weinberg equilibrium (HWE) on the genotype distribution of the* ACTN3* gene and establish whether the sample was from a normally distributed population. One-way analyses of variance followed by Tukey post hoc tests, if necessary, were used to test significant differences in dependent variables across the* ACTN3* genotypes. With respect to BMDs of lumbar spine (L1–L4), femur (neck and total), and total body, the analyses were performed for each variable in duplicate in the units of g/cm^2^ and *T*-score. The overall significance level was set to alpha 0.05. To correct for multiple testing, therefore, we used the* Bonferroni* method to obtain the significance level (*p* = 0.025) used for every test.

Genotypes were coded 0 = RR, 1 = RX, and 2 = XX for examination of an additive genotype effect. Genotype groups were collapsed to test dominant (any X versus RR) and recessive (XX versus any R) effects. Associations with osteoporotic status and sarcopenia were examined by binary logistic regressions, with correction for measured covariates. Results were expressed as odds ratio (OR) and 95% confidence interval (95% CI) with reference to normal group with respect to sarcopenia and osteoporotic status (osteopenia plus osteoporosis), respectively. Statistical significance was set at *p* = 0.05. All statistical analyses were performed using SPSS version 20.0 software.

## 3. Results

The mutant genotype of the* ACTN3* gene (XX) was found in 20.8% of 332 study participants. The ancestral genotypes were 26.5% for homozygous (RR) and 52.7% for heterozygous (RX). The genotype frequencies for the* ACTN3* gene were in HWE (*p* = 0.293) (Table 1). There was no significant gender difference (*χ*^2^ = 1.879, *p* = 0.391) in XX (14.5% and 22.2% for men and women, resp.) or RR (27.4% and 26.3% for men and women, resp.) or RX (58.1% and 51.5% for men and women, resp.) genotype. No significant differences were observed in mean age (*p* = 0.716), height (*p* = 0.150), weight (*p* = 0.067), BMI (*p* = 0.456), or body fat percentage (*p* = 0.831) across the genotypes (Table 2).

In this study population, we found that prevalence of sarcopenia was 46.8% in men (mean aged 74.4 ± 4.6 years) and 23.3% in women (mean age of 74.4 ± 6.6 years) (data not provided). Prevalence of sarcopenia for men was similar to that of a previous study done by Kim et al. [31], whereas prevalence of sarcopenia for women was higher than that of the same study and similar to that of another study done by Kwon et al. [32]. We suspect that this discrepancy results from the differences in general nutritional status and environments as well as its definitions used. In addition, prevalence of osteopenia was 34.5% in men (mean age of 73.3 ± 5.7 years) and 58.5% in women (mean age of 73.3 ± 6.0 years), and prevalence of osteoporosis was 0% in men and 25.9% in women (mean age of 77.7 ± 6.0 years) (data not provided). Analyzing the data obtained from the 2008–2011 in Korea National Health and Nutrition Examination Survey (KNHANES) IV and V, Park et al. [33] reported that prevalence of osteopenia in Korea was 46.5% in men and 48.7% in women aged 50 years and older, and prevalence of osteoporosis in Korea was 7.3% in men and 38.0% in women aged 50 years and older. We suggest that this discrepancy results from the differences in general nutritional status, environments (such as sun exposure and daily physical activities) according to each residential area, and others. This difference may also result from the fact that *T*-scores are calculated using different normal reference data. Our study used a manufacturer-provided reference population based on a healthy Korean-adult matched for age and sex in Korean general population aged 20–40 years old (from the manufacturer's technical data).

With respect to bone health, significant differences in ACTN3 genotypes were seen in relative BMDs for femur neck (*p* = 0.007) and femur total (*p* = 0.022) and in *T*-scores for femur neck (*p* = 0.007) and total body (*p* = 0.017), with similar trends in femur total (*p* = 0.022) and total body (*p* = 0.030) across the genotypes (Table 3). Tukey post hoc tests showed that RR homozygotes and RX heterozygotes had significantly higher BMDs for femur neck (*p* = 0.005 and *p* = 0.050, resp.) than XX homozygotes, with no such difference between RX and XX genotypes. RR homozygotes had a significantly higher BMD for femur total (*p* = 0.018) than XX homozygotes, with no such difference between RR and RX genotypes or between RX and XX genotypes. Similarly, post hoc tests showed that RR homozygotes and RX heterozygotes had significantly higher *T*-scores for femur neck (*p* = 0.006 and *p* = 0.042, resp.) than XX homozygotes, with no such difference between RR and RX genotypes. In addition, RR homozygotes and RX heterozygotes had significantly higher *T*-scores for total body (*p* = 0.019 and 0.036, resp.) than XX homozygotes, with no such difference between XX and RX genotypes. With respect to sarcopenia, there was a significant difference (*p* = 0.016) in ASM index across ACTN3 genotypes. Tukey post hoc test showed that RR homozygotes had a significantly higher ASM than XX homozygotes (*p* = 0.012), with no such difference between RR and RX genotypes or between RX and XX genotypes. No significant differences were found in serum vitamin D levels across ACTN3 genotypes.

Binary logistic regression analyses showed that sarcopenia was significantly associated with the presence of one (OR = 2.020, 95%  CI = 1.045–3.904, *p* = 0.037) or two copies (OR = 2.237, 95%  CI = 1.044–4.836, *p* = 0.038) of 577X, independent of the covariates (Table 4). Under a dominant model, this result was significant (OR = 2.056, 95%  CI = 1.024–4.127, *p* = 0.043) only when unadjusted for the covariates. Under a recessive model, however, the risk of 577X to sarcopenia remained significant (OR = 1.970, 95%  CI = 1.070–3.629, *p* = 0.029) even when adjusted for the covariates. Osteoporotic status was significantly associated with the presence of one (OR = 2.305, 95%  CI = 1.057–5.024, *p* = 0.036) and two copies (OR = 2.794, 95%  CI = 1.208–5.461, *p* = 0.016) of 577X. The ORs were not significant (*p* = 0.130 and *p* = 0.075 for RX and XX, resp.) when adjusted for the covariates (Table 5). Under a dominant model, the result was not significantly regardless of the covariates. Under a recessive model, however, the result was significant (OR = 2.463, 95%  CI = 1.160–5.227, *p* = 0.019), which was no longer significant (*p* = 0.319) when adjusted for the covariates.

## 4. Discussion

With advancing aging, loss of LBM and BMD represents a big threat to loss of independence in later life in Korea. Considering the fact that individual variability of LBM and BMD is primarily determined by genetic makeup [8, 9], determining the molecular factors that contribute to declines of LBM and BMD with aging is important and clinically relevant. In this study, therefore, we examined the susceptibility of* ACTN3* R577X genotype to sarcopenia and osteoporotic status in a sample of Korean older adults and found the detrimental influence of the X allele on LBM and BMD. X allele carriers under general, dominant, and recessive models had a greater risk of sarcopenia than R alleles. Additionally, X allele carriers under general and recessive models had a greater risk of low BMD than R allele carriers. In particular, the increased risk of X alleles for sarcopenia remained significant under general and recessive models even after adjustments for age, gender, BMI, and percent body fat, whereas the increased risk of XX homozygotes for osteoporotic status was significantly modulated by variability of the covariates.

Findings of this study were in agreement with previous studies reporting predisposition of people with the X allele to impaired muscle strength, power, and physical function. For example, Zempo et al. [34] showed that older Japanese women who are XX homozygotes have lower thigh muscle mass than RR homozygotes or RX heterozygotes. Using data on women from the Baltimore Longitudinal Study of Aging, Walsh et al. [26] reported that XX homozygotes had lower values for total body and lower limb FFMs than RR homozygotes or RX heterozygotes. Using two large cohorts of Caucasian postmenopausal women, Judson et al. [35] found that X allele carriers (i.e., XX homozygotes or RX heterozygotes) had a greater risk of falling than RR homozygotes. In addition, the XX alleles were positively associated with exertional rhabdomyolysis [36] and inflammatory myopathy cases [37]. Similar to those clinical studies,* Actn3* KO mice were found to have lower muscle mass [17], smaller fast-twitch fiber diameter [17, 18], and greater decline of quadriceps mass [19], possibly due to calcineurin signaling-induced activation of the slow myogenic program toward a slow-twitch and oxidative phenotype [20], compared to wild-type mice. Taken together, those findings including the current one suggest that the* ACTN3* R577X genotype may contribute to changes in muscle mass and/strength with aging.

With respect to elderly Koreans, there is one previous study reporting an association between the* ACTN3* genotype and low BMDs. Min et al. [22] reported that carriers of the RR genotype tended to have greater BMDs than carriers of either the RX or XX genotypes in older Korean women. Their study has low statistical power due to a small sample size (*n* = 68), reducing a chance of detecting a true effect. In addition, they did not include sarcopenia as an outcome variable. However, the current study extends the previous finding by reporting that the* ACTN3* genotype is significantly associated with variability of osteoporotic status as well as sarcopenia in a relatively large sample size of Korean older adults. In addition, the current findings show that the susceptibility of the X allele to sarcopenia and bone health may be somewhat modulated by modifiable risk factors such as body fatness and serum vitamin D levels, implying the significance of a healthy lifestyle consisting of a good nutrition and physical activity in minimizing the susceptibility of the ACTN3 genotype to the detrimental health consequences with aging.

Several explanations are possible for the protective role of the R allele against loss of LBM and BMD. The R allele may function against muscle atrophy with sedentary aging, since it is associated with higher testosterone levels [38] in Russian athletes, increased muscle mass [39], and increased proportion of fast-twitch muscle fibers [40]. On the other hand, homozygosity for the X allele results in complete loss of function and absence of *α*-actinin-3 protein [41], which may be detrimental to optimal function of muscle strength/power [42] and beneficial to endurance performance [43]. Using microarray data analysis, Yang et al. [23] showed that* Actn3* KO mice have altered expression of several genes regulating bone mass and osteoblast/osteoclast activity, contributing to the regulation of bone mass through alterations in bone turnover. In addition, growing evidence shows that the* ACTN3* gene X allele is associated with sensitivity to reduced anabolic hormone secretion [38], anabolic or catabolic molecules released by skeletal muscle or by bone cells (myokines and osteokines) [20, 23], reduced physical activity/fitness [44], and altered osteoblast/osteoclast activity leading to decreased BMD [23, 45]. Collectively, these previous findings suggest that the* ACTN3* gene X allele may share common pathways mediating susceptibility to sarcopenia and osteoporosis associated with aging. Yet, the biological mechanism(s) by which the ACTN3 R557X SNP contributes to loss of BMD with aging remains to be further investigated.

This study has some limitations. First, a cause-effect relationship of the* ACTN3* genotype and sarcopenia and osteoporosis could not be determined due to the cross-sectional nature of the study. Second, the* ACTN3* R577X polymorphism and its association with sarcopenia may be gender-dependent. Our data were not analyzed in women and men separately because we did not have sufficient power to test the hypothesis. Therefore, whether gender modulates the association between the* ACTN3* genotype and sarcopenia and osteoporosis remains to be determined. Third, lifestyle risk factors such as physical inactivity, poor fitness, and malnutrition may be significant modulators in determining the susceptibility of people with the* ACTN3 *genotype to sarcopenia and osteoporosis. Lastly,* ACTN3* gene is one of the candidate genes contributing to variations in muscle and bone health phenotypes. The current findings need to be confirmed in a future study using several variants and a larger sample size.

## 5. Conclusion

In summary, we examined the association between the* ACTN3* R577X polymorphism and sarcopenia and osteoporotic status in older Korean adults and found increased risk for sarcopenia and osteoporosis with the X allele. Future studies should investigate the extent to which the* ACTN3* R577X polymorphism affects alterations in LBM and BMD with aging and the mechanisms by which* ACTN3* deficiency interacts with modifiable risk factors such as malnutrition and physical inactivity to influence loss of LBM and BMD in older adults.

## Figures and Tables

**Table 1:** Genotype distribution of *ACTN3 *gene in the study population.

	Genotype frequency, *N* (%)			Allele frequency, *N* (%)	
	RR	RX	XX	R	X
Subjects (*N* = 332)	88 (26.5)	175 (52.7)	69 (20.8)	175.5 (52.86)	156.5 (47.13)
HWE	92.77 (27.94)	165.46 (49.84)	73.77 (22.22)		

HWE: Hardy-Weinberg equilibrium; *p* = 0.293.

**Table 2:** Physical and bone health characteristics of study participants according to *ACTN3* genotypes.

Variable	Genotype			*p* value
	RR (*n* = 88)	RX (*n* = 175)	XX (*n* = 69)	
Age (years)	72.9 ± 7.0	73.8 ± 6.5	74.6 ± 6.3	0.716
Height (cm)	155.4 ± 7.4	154.7 ± 7.4	153.1 ± 6.4	0.150
Weight (kg)	58.7 ± 7.9	58.5 ± 8.8	55.9 ± 7.9	0.067
BMI (kg/m^2^)	24.3 ± 2.8	24.5 ± 3.4	23.9 ± 2.7	0.456
Body fatness (%)	33.6 ± 8.1	34.1 ± 8.0	34.2 ± 6.1	0.831

BMI: body mass index.

**Table 3:** Measured parameters of bone mineral density and sarcopenia according to the *ACTN3* genotypes.

	ACTN3 genotype			*p* values
	RR (*n* = 88)	RX (*n* = 175)	XX (*n* = 69)	
Sites of BMD				
L1–L4 (g/cm^2^)	0.991 ± 0.186	0.984 ± 0.167	0.947 ± 0.167	0.254
L1–L4 *T*-score	−1.619 ± 1.175	−1.478 ± 1.269	−1.784 ± 1.220	0.232
L2–L4 (g/cm^2^)	1.024 ± 0.196	1.008 ± 0.172	0.967 ± 0.173	0.140
L2–L4 *T*-score	−1.249 ± 1.601	−1.386 ± 1.419	−1.720 ± 1.431	0.142
Femur neck (g/cm^2^)	0.764 ± 0.134	0.742 ± 0.124	0.701 ± 0.110^*∗*^	0.007
Femur neck *T*-score	−1.499 ± 1.085	−1.658 ± 1.018	−2.010 ± 0.914^*∗*,#^	0.007
Femur total (g/cm^2^)	0.847 ± 0.134	0.828 ± 0.132	0.788 ± 0.133^*∗*^	0.022
Femur total *T*-score	−1.019 ± 1.166	−1.158 ± 1.132	−1.507 ± 1.148	0.026
Total body (g/cm^2^)	1.005 ± 0.106	0.999 ± 0.100	0.965 ± 0.104	0.030
Total body *T* score	−1.375 ± 1.121	−1.466 ± 1.071	−1.851 ± 1.108^*∗*,#^	0.017
ASM (kg/m^2^)	6.175 ± 0.846	6.049 ± 0.783	5.821 ± 0.601^*∗*^	0.016
Vitamin D (nmol/L)	46.7 ± 26.5	45.9 ± 23.5	44.9 ± 19.0	0.368

BMD: bone mass density; L: lumbar; ASM: appendicular skeletal muscle mass; the Bonferroni critical value (*p* = 0.025) was used for statistical significances of BMDs and *T*-scores by dividing *p* = 0.05 by the number of tests (*n* = 2).

*∗* denotes that XX homozygotes were significantly different compared to RR homozygotes. # denotes that XX homozygotes were significantly different compared to RX heterozygotes.

**Table 4:** Odds ratio (OR) and 95% confidence interval (95% CI) of *ACTN3* genotype for sarcopenia.

Genotype	OR_1_ (95% CI)	*p*_1_	OR_2_ (95% CI)	*p*_2_
RR versus RX	1.212 (0.664–2.213)	0.530	1.136 (0.595–2.168)	0.699
RX versus XX	1.696 (.938–3.065)	0.080	2.020 (1.045–3.904)	0.037
RR versus XX	2.056 (1.024–4.127)	0.043	2.237 (1.044–4.836)	0.038
Dominant	2.056 (1.024–4.127)	0.043	1.401 (0.760–2.582)	0.279
Recessive	2.056 (1.024–4.127)	0.043	1.970 (1.070–3.629)	0.029

CI: confidence interval; OR: odds ratio; dominant: RR versus RX + XX; recessive: RR + RX versus XX; OR_1_ and *p*_1_: unadjusted; OR_2_ and *p*_2_ are adjusted for age, gender, body fatness, and vitamin D.

**Table 5:** Odds ratio (OR) and 95% confidence interval (95% CI) of *ACTN3* genotype for osteoporotic status.

Genotype	OR_1_ (95% CI)	*p*_1_	OR_2_ (95% CI)	*p*_2_
RR versus RX	1.212 (0.685–2.146)	0.509	1.314 (0.657–2.627)	0.441
RX versus XX	2.305 (1.057–5.024)	0.036	1.969 (0.819–4.735)	0.130
RR versus XX	2.794 (1.208–5.461)	0.016	2.682 (0.960–7.942)	0.075
Dominant	1.476 (0.852–2.558)	0.165	1.492 (0.769–2.893)	0.237
Recessive	2.463 (1.160–5.227)	0.019	2.158 (0.891–5.224)	0.319

CI: confidence interval; OR: odds ratio; dominant: RR versus RX + XX; recessive: RR + RX versus XX; OR_1_ and *p*_1_: unadjusted; OR_2_ and *p*_2_ are adjusted for age, gender, body fatness, and vitamin D.
